# Evaluating of Life Quality in Patients with Acne Vulgaris Using Generic and Specific Questionnaires

**DOI:** 10.1155/2013/108624

**Published:** 2013-11-25

**Authors:** Reza Ghaderi, Alireza Saadatjoo, Faezeh Ghaderi

**Affiliations:** ^1^Department of Dermatology, Birjand University of Medical Sciences, Ghaffari Street, Birjand 9717735338, Iran; ^2^Faculty of Nursing, Birjand University of Medical Sciences, Ghaffari Street, Birjand 9717735338, Iran; ^3^Department of Paediatric Dentistry, Shiraz University of Medical Sciences, Dental School, Ghasrodasht Street, Shiraz 7196515878, Iran

## Abstract

*Background*. Acne vulgaris is a common skin disease that can adversely affect the quality of life of patients. *Objective*. The aim of this study was to determine the quality of life in patients with acne vulgaris. *Methods*. This study was carried out on 70 patients with acne vulgaris (28 males, 42 females). All the patients filled out two Persian versions of questionnaires: short form 36 (SF-36) and Dermatology Life Quality Index (DLQI). The obtained data were analyzed by using SPSS software (version 17). *Results*. The scores for physical functioning, social functioning, and bodily pain domains in patients were over 70%, but the scores for role physical, general health, vitality, role emotional, and mental health in patients were under 70%. Scores on the DLQI in patients with acne vulgaris ranged from 0 to 22 (mean ± SD, 8.18 ± 4.83). After comparing mean score of DLQI with respect to gender and age, it was found that the difference between the two groups was not statistically significant. *Conclusion*. Acne vulgaris has a significant effect on the quality of life. There was not any significant gender or age related difference in QOL.

## 1. Introduction

 Quality of life is a general term which includes a feeling of joy and satisfaction with life. Quality of life (QOL), self confidences, and self esteem in patients with skin diseases have not sufficiently been attended to. Since skin diseases affect well-being, general health, function, and social adaptation of the individual, they can decrease self confidence of the patient and definitely disrupt self image or cutaneous body image, mental health, and quality of his life [1, 2]. By even having chronic itching, life of a person will be charily disturbed [3].

 Besides evaluating the treating procedure, recording QOL can advance our knowledge regarding psychosocial stress associated with dermatologic disorders [4]. 

 According to the studies carried out in Canada [5], USA [6], Egypt [7], Denmark [8], Iran [9], UK [10], Turkey [11], Saudi Arabia [12], Brazil [13], and many other countries [14–19], skin diseases have a great effect on the QOL of patients.

Patient's life would be influenced by acne vulgaris both physically and emotionally [15].

 Psychological involvements include anxiety, depression, and those which deeply affect life's quality [16, 17]. 

 The present study can declare the importance of psychological aspects to its readers to the extent that psychological consultation and intervention may be in regard to patients with acne vulgaris. Moreover, measuring the incapacities of patients with the aim of taking better care of them can be helpful in leading health-care services towards the real needs of the patients.

 Considering the above and the fact that according to WHO's programs one of the duties of health-care officials is to promote the level of QOL, the present study was conducted to evaluate QOL in Iranian patients with acne vulgaris.

## 2. Methods

This study was performed on 70 patients with acne vulgaris. Inclusion criteria include having acne vulgaris and being over the 16 years. Exclusion criteria include having skin diseases different from the acne vulgaris, having a chronic disease which would have an effect on QOL, or having apparent disability. The study was carried out in the dermatology outpatient clinic of Valiyy-e-asr hospital in Birjand, Iran, between May 2009 and May 2010. All those who had referred to the clinic had the entrance requisites for the study.

The aims of the survey were explained to them. The responders gave their informed written consent to participate in the study. The institutional ethical committee approved the study protocol.

 Two Persian versions of questionnaires, DLQI (which is a specific questionnaire) and SF-36 which, is a generic one, were given to all those diagnosed with acne vulgaris by a dermatologist.

DLQI questionnaire was designed by Finlay and Khan in 1992, and since then, it has vastly been used in different communities [1, 7, 9–11, 13, 14, 16, 18].

Furthermore, reliability and validity of the Persian version of the DLQI questionnaire had been proved through a study in a group of Iranian patients with vitiligo in Shiraz by Aghaei et al. [9]. Reliability analysis showed satisfactory result (Cronbach's alpha coefficient = 0.77) [9].

The questionnaire includes 10 multiple-choice questions; the points for each question range between “0” and “3.” Total score of every individual's QOL would be the sum of total scores of all the questions, that is, between 0 and 30; the more an individual's score, the worse his QOL. The questionnaire was divided into 6 parts: symptoms and feelings (questions 1 and 2), daily routine activities (questions 3 and 4), leisure and spare time (questions 5 and 6), individual relations (questions 8 and 9) occupation and school (question 7), and treatment (question 10) [10].

SF-36 questionnaire is a generic one which has vastly been used in different communities [12, 13, 19, 20, 31–35]. Furthermore, reliability and validity of the Persian version of SF-36 questionnaire had been proved through a study in Tehran by Montazeri et al. [33] and another study in Shiraz by Jafari et al. [34]. Reliability analysis showed satisfactory results (Cronbach's alpha coefficient = 0.915) [34].

 “It Comprises of 36 items assessing eight domains of life quality: Physical Functioning (PF); Role Physical (RP), which refers to role restrictions due to physical problems; Bodily Pain (BP); General Health(GH); Vitality (VT); Social Functioning (SF); Role Emotional (RE), which refers to role restrictions due to emotional problems; and Mental Health (MH)” [12]. The achieved scores in each of the domains are separately summed up ranging from “0” to “100”; the more an individual's score, the better his QOL. Data were analyzed by SPSS software (version 17). Students *t*-test, analysis of variance (ANOVA), Tukey ranged tests and Pearson's correlation coefficient test were properly applied for comparison of means. Statistical significance was defined at *P* < 0.05.

## 3. Results

Seventy patients diagnosed with acne vulgaris by a dermatologist were included in this study. Twenty-eight (40%) of the patients were males and 42 (60%) females. 

The age of patients ranged from 16 to 34 years (mean ± SD, 21.89 ± 4.62). As to occupation, most of patients were university or high-school students (29 = 41.4%), and some were clerks (16 = 22.85%), whereas the others were housewives (6 = 8.6%), farmers (3 = 4.3%), or unemployed (16 = 22.85%). 

### 3.1. SF-36

The scores for physical functioning, social functioning, and bodily pain domains in patients were over 70%, but the scores for role physical, general health, vitality, role emotional and mental health in patients were under 70% (Table 1).

After comparing mean score of QOL (SF-36) with respect to gender, the difference between men's scores and women's scores was found but it was not statistically significant (*P* = 0.70) (Table 1).

Comparing mean total score of QOL (SF-36) in regard to age using Tukey ranged test and ANOVA indicated that the mean score was different in different age groups but the difference was not significant. (ANOVA, *P* = 0.780) (Table 2).

### 3.2. DLQI

Scores on the DLQI in patients with acne vulgaris ranged from 0 to 22 (mean ± SD, 8.18 ± 4.83). The mean scores of DLQI in male and female were 8.75 (±4.71) and 7.93 (±4.91), which was not statistically significant (*t* = 0.697, df = 68, *P* = 0.488). The mean score of DLQI in regard to age was distinct in different age groups, but the difference was not significant. (*P* = 0.51) (Table 3).

Pearson's correlation coefficient test revealed that there was a reverse and significant correlation between all the domains of QOL in SF-36 questionnaire and the obtained scores from DLQI questionnaire. In other words, the higher the QOL score in any of the dimensions (SF-36), the lower the DLQI score and vice versa (Table 4).

## 4. Discussion

Acne vulgaris is a very frequent skin disease that mostly involves adults both physically and emotionally, while it can also influence individuals in any age [12, 15, 21].

Acne has remarkable effect on self image and influences health-related quality of life (HRQL) [2, 19, 20, 22, 36] (Table 5). 

### 4.1. SF-36

Our findings somewhat correlate with other studies evaluating acne patients' quality of life [12, 20, 22, 23, 37]. 

Al Robaee found that for recorded scores physical functioning, role physical, role emotional, and vitality dimension (SF-36) in patients with acne vulgaris were under 60% [12]. 

In some of previous studies, there was not a clear-cut correlation between life quality score and gender [22], and it agrees with our finding. But this is not in accordance with what Al Robaee found that female patients with acne were more probably to express better general health than males [12]. This difference seen between our finding and Al Robaee's might be due to cultural or socioeconomic differences.

On the other hand, Tan et al. showed that greater effect on life quality was related with older age, female sex [5]. This difference seen between our finding and the Tan et al. one can be due to their study limitations as they reported (include (1) patients with acne were referred from the other centers, no first hand patients, (2) the limitation of final result values to facial acne).

### 4.2. DLQI

The mean DLQI score in the patients of this study is higher compared to that in the studies by Takahashi et al. (mean DLQI = 3.99) in Japan (Tokyo) [23]. This difference seen between the result of the present study and other study can be due to cultural or socioeconomic differences.

Generally, in most of previous studies, there was not a correlation between DLQI score and gender [14, 23] which is consistent with our finding. But these results disagree with Abdel-Hafez et al. [7] who found a correlation between DLQI score and gender. They reported that mean DLQI scores of male with acne are higher than those of female patients [7]. This result disagrees with our result. This difference seen between the result of the present study and other study can be due to duration and severity of acne differences.

Comparing mean score of DLQI in regard to age in our study is in accordinate with the finding by Takahashi et al. [23] but disagrees with Lasek et al. They performed the study in USA and found that patients aged 40 years or older expressed greater impacts of their acne on their life quality than junior patients [24]. The difference seen between our finding and other study can be due to older age of their patients (mean  age ± SD was 31 ± 10.1 years).

### 4.3. Relationship between Acne's Quality of Life and Treatment

Recent results recommended that the treatment strategies to reduce the number and size of lesions of acne vulgaris can improve their life quality [16–18, 25–27, 36, 38].

Ammad et al. studied the employment of intense blue light with spectrum ranging 415–425 nm (peak 420 nm) in the treatment of acne vulgaris. Significant improvement was obtained in the DLQI after the treatment [38].

 Matsuoka et al. showed that directions on the usage of skin care and cosmetics for female patients with acne did not worsen acne treatment and affected patients' life quality effectively. They suggested program for using skin care and cosmetics supplement traditional medical therapeutics for acne [26].

Fried and Nighland found that prescription of tretinoin gel microsphere in acne vulgaris patients resulted in a significant increase in both life quality and patient satisfaction [27].

Kobayashi et al. found that roxithromycin has a therapeutic effect on inflammatory acne and leads to enhancement of life quality in the patients [18].

Hahm et al. indicated that oral administration of isotretinoin in patients with acne vulgaris relieved symptoms of depression which was mostly related to acne-related life quality enhancements rather than to improvement in acne grade [39]. Besides, Niemeier et al. suggested that dermatologists should have some knowledge of the basics of psychotherapy and psychopharmacology, which sometimes must be combined with systemic and topical treatment of acne in conjunction with basic psychosomatic treatment [17]. 

On the hand, adherence to treatment is an important issue in dermatology [28, 29]. Poor designed program in treatment sequel may be a popular reason of treatment failure in patients with acne [30]. 

Jones-Caballero et al. stated that dermatologists should clarify that following and support in treatment sequels directly related to the better results and better life quality [29]. 

Lott et al. found a positive correlation between life quality of patients with acne and obedience in treatment. The reason for this failure in treatment has been reported to be shortened time of the patients in a daily life [30].

Based on these findings, dermatologists should consider medication adherence as an important factor in further dermatologic investigations.

Regarding the findings of related studies in this issue, it seems more advisable that psychiatric consult or psychotherapy may also be included in the treatment of severe acne vulgaris. Besides, to promote patients' satisfaction and their quality of life, the following points are recommended.

1. Setting up supportive groups to patients in the specialized dermatology departments and hospitals.
2. Forming consultation and psychotherapy centers for patients with severe acne vulgaris.
3. Dermatologists should consider the impact of acne vulgaris on health-related quality of life and educate patients on possible treatments.
4. Dermatologists should consider an early and effective therapy especially in patients with severe acne vulgaris to better medication adherence.

## 5. Limitation

Limitation in our study was that all patients had referred to the clinic; thus, we do not know what impact acne has on QOL in people with acne who do not choose to or cannot come to the doctor.

## 6. Conclusion

Acne vulgaris has a significant effect on the quality of life. There was not any significant gender or age related difference in QOL.

## Figures and Tables

**Table 1:** Comparison of mean score of QOL (SF-36) in different domains with respect to gender using *t*-test.

Domains	Male (*n* = 28)	Female (*n* = 42)	Total	*P* value
	Mean ± std. deviation	Mean ± std. deviation	Mean ± std. deviation	
PF	75.71 ± 23.40	78.81 ± 23.13	77.57 ± 23.12	0.59
RP	61.61 ± 32.26	73.81 ± 31.70	68.93 ± 32.26	0.12
RE	53.57 ± 39.90	60.32 ± 34.72	57.62 ± 36.74	0.46
VT	62.32 ± 17.77	60.59 ± 21.93	61.29 ± 20.25	0.73
MH	60.00 ± 16.07	56.57 ± 21.70	57.94 ± 19.59	0.48
SF	81.70 ± 19.39	74.11 ± 18.60	77.14 ± 19.15	0.11
BP	78.57 ± 19.89	78.27 ± 19.58	78.39 ± 19.56	0.95
GH	59.82 ± 17.82	63.18 ± 16.23	61.84 ± 16.84	0.42
Total	66.66 ± 15.66	68.21 ± 16.49	67.59 ± 16.07	0.70

Physical functioning (PF); role physical (RP); role emotional (RE); Vitality (VT); mental health (MH); social functioning (SF); bodily pain (BP); general health (GH).

**Table 2:** Comparison of mean score of QOL (SF36) in different domains in patients with acne vulgaris with respect to age using ANOVA.

Domains	<20	20–30	>30	*P* value
	Mean ± std. deviation	Mean ± std. deviation	Mean ± std. deviation	
PF	75.18 ± 25.13	77.56 ± 22.44	93.75 ± 6.29	0.33
RP	71.29 ± 29.98	66.02 ± 34.64	81.25 ± 23.93	0.60
RE	56.79 ± 39.02	58.11 ± 36.44	58.33 ± 31.91	0.99
VT	62.96 ± 17.50	59.61 ± 22.51	66.25 ± 16.00	0.71
MH	58.96 ± 18.48	56.92 ± 20.79	61.00 ± 18.86	0.87
SF	81.01 ± 20.02	75.32 ± 19.12	68.75 ± 7.21	0.33
BP	82.68 ± 15.11	75.76 ± 22.36	75.00 ± 14.57	0.35
GH	60.50 ± 15.34	62.43 ± 18.63	65.00 ± 7.07	0.84
Total	68.67 ± 15.02	66.47 ± 17.39	71.17 ± 10.21	0.78

Physical functioning (PF); role physical (RP); role emotional (RE); Vitality (VT); mental health (MH); social functioning (SF); bodily pain (BP); general health (GH).

**Table 3:** Comparison of mean score of DLQI in patients with acne vulgaris regard to age using ANOVA.

Age	Mean	Std. deviation	*P* value
<20	8.07	5.28	0.51
20–30	8.64	4.69	
>30	5.75	1.26	

**Table 4:** Pearson correlation between the scores of different domains of QOL obtained from the two questionnaires (SF-36 and DLQI) in patients with acne vulgaris.

	PF	RP	RE	VT	MH	SF	BP	GH	Total
DLQI	*r* = −0.20	*r* = −0.37	*r* = −0.49	*r* = −0.36	*r* = −0.19	*r* = −0.21	*r* = −0.31	*r* = −0.38	*r* = −0.46
	*P* = 0.10	*P* = 0.002	*P* = 0.000	*P* = 0.002	*P* = 0.11	*P* = 0.09	*P* = 0.009	*P* = 0.001	*P* = 0.000

Physical functioning (PF); role physical (RP); role emotional (RE); vitality (VT); mental health (MH); social functioning (SF); bodily pain (BP); general health (GH).

**Table 5:** The summary of previous studies that looked at QOL in acne.

Author	Questionnaire	Year	Country	Reference
Tan et al.	SF36	2008	Japan	[5]
Abdel-Hafez et al.	DLQI	2009	Egypt	[7]
Al Robaee	SF36	2009	Saudi Arabia	[12]
Kaymak et al.	DLQI	2009	Palestine	[16]
Kobayashi et al.	DLQI & kindex-29-J	2009	Japan	[18]
Arslan et al.	SF36	2009	Turkey	[20]
Do et al.	The Self Image & Rosenberg Self-Esteem	2009	Korea	[21]
Mallon et al.	Rosenberg Self-Esteem & General Health & DLQI & SF36	1999	UK	[22]
Takahashi et al.	DLQI	2006	Japan	[23]
Lasek and Chren	DLQI	1998	USA	[24]
Jones-Caballero et al.	Skindex-29	2007	Spain	[25]
Matsuoka et al.	WHOQOL26 & DLQI	2006	Japan	[26]
Fried and Nighland	Acne Quality of Life Index	2009	USA	[27]
Dréno	Reviews the different scales	2006	France	[28]
Jones-Caballero et al.	Skindex-29	2008	Spain	[29]
Lott et al.	Review	2010	USA	[30]
